# Clinical features and prognostic analysis of 120 patients with oropharyngeal squamous cell carcinoma: a hospital-based real-world study

**DOI:** 10.3389/fonc.2025.1533688

**Published:** 2025-05-30

**Authors:** Li Zhang, Rui Wang, Jing Wang, Dapeng Wang, Feifei Ma, Zhilin Li, Shuxin Wen

**Affiliations:** ^1^ Shanxi Medical University, Taiyuan, China; ^2^ Department of Head and Neck Surgery, Shanxi Province Cancer Hospital, Shanxi Hospital Affiliated to Cancer Hospital, Chinese Academy of Medical Sciences, Cancer Hospital Affiliated to Shanxi Medical University, Taiyuan, China; ^3^ Department of Otolaryngology Head and Neck Surgery, Third Hospital of Shanxi Medical University, Taiyuan, China; ^4^ Department of Otolaryngology Head and Neck Surgery, Shanxi Bethune Hospital, Taiyuan, Shanxi, China; ^5^ Department of Pathology, Shanxi Province Cancer Hospital, Taiyuan, China; ^6^ Department of Radiation Oncology, Shanxi Province Cancer Hospital, Taiyuan, China; ^7^ Department of Nephrology, Shanxi Provincial People’s Hospital, Taiyuan, Shanxi, China

**Keywords:** oropharyngeal squamous cell carcinoma, HPV, p16, sub-anatomical sites, surgical treatment, prognostic

## Abstract

**Objective:**

To investigate the clinical characteristics and current treatment status of oropharyngeal squamous cell carcinoma (OPSCC) patients in Shanxi Province, China, and to examine the relationship between these factors and human papillomavirus (HPV) status, as well as identify risk factors influencing prognosis.

**Methods:**

We retrospectively analyzed the medical records of 120 OPSCC patients from two tertiary hospitals in Shanxi Province. Statistical analyses were performed to assess the relationship between various clinicopathological factors, treatment modalities, and p16 status, as well as their impact on patient prognosis.

**Results:**

The most common sub-anatomical sites of OPSCC were the tonsils and the base of the tongue, with a significantly higher proportion of p16-positive cases compared to other sub-sites (P = 0.033). The majority of cases were poorly differentiated squamous cell carcinoma (63 cases, 52.5%), of which 71.4% were p16-positive (P = 0.002). Patients with p16-positive OPSCC were more likely to present with a neck mass as the initial symptom (73.2%, P = 0.019). Overall, p16-positive OPSCC patients had a better prognosis (P = 0.008); however, p16-positive patients with primary tumors located in the soft palate and posterior pharyngeal wall did not show a significant prognostic advantage compared to p16-negative patients. Surgical treatment did not improve survival rates for OPSCC patients, particularly in the p16-positive group, where the survival curves showed significant separation approximately one year after treatment, indicating better outcomes in the non-surgical group.

**Conclusion:**

In North China’s Shanxi Province, the incidence of HPV-associated OPSCC has surpassed that of OPSCC caused by smoking and alcohol use. p16-positive patients with primary tumors located in the soft palate and posterior pharyngeal wall have a poor prognosis, indicating that treatment de-escalation should be approached with caution. Traditional open surgical treatment, without consideration of HPV status, does not appear to benefit patients.

## Highlights

- The incidence of HPV-related OPSCC has reached 57.5% in North China’s Shanxi Province.
- Not all HPV-positive OPSCC cases exhibit favorable prognosis.
- Traditional open surgery, regardless of HPV status, does not benefit patients.

## 1 Background

In recent years, the proportion of human papillomavirus-associated oropharyngeal squamous cell carcinoma (HPV-OPSCC) among head and neck squamous cell carcinomas has significantly increased, leading to a rising incidence of OPSCC globally, with over 100,000 cases reported in 2020 (1). There is a marked variation in HPV infection rates among OPSCC patients in different countries. A 2021 study (2) reported a global HPV positivity rate of 31% among OPSCC patients, with rates ranging from 0% to 85% depending on the geographic region, and higher rates were observed in economically developed regions—for instance, the proportion of HPV-positive OPSCC cases in the United States had already increased to 75% by 2015.

Due to the significantly better prognosis associated with HPV-OPSCC, the latest American Joint Committee on Cancer (AJCC) staging system for oropharyngeal cancer now includes HPV tumor status, which can significantly reduce the tumor stage. Several clinical trials (3–6) are actively exploring treatment de-escalation approaches. However, as research progresses, some scholars (7) have found that not all HPV-OPSCC cases have favorable outcomes, with approximately 17.3% of HPV-OPSCC patients experiencing locoregional recurrence and 6.5% developing distant metastases within three years after treatment. Another study (8) showed that up to 5% of HPV-OPSCC patients experience persistent disease progression despite receiving curative treatment, leading to poor prognosis.

It is important to note that the role of HPV in OPSCC varies significantly depending on geographic, economic, and cultural differences. Most related studies have focused on developed Western countries, with fewer studies conducted in China. The epidemiology, clinical characteristics, and prognosis of HPV-positive OPSCC patients in the real-world setting of Shanxi, an economically underdeveloped region in North China, remain unclear. This issue warrants further investigation to address existing knowledge gaps and to provide evidence-based support for the development of precise diagnostic and treatment strategies for OPSCC across different regions of China. It holds substantial practical significance and broad applicability.

## 2 Materials and methods

### 2.1 General information

#### 2.1.1 Study population

This study included 120 hospitalized patients with pathologically confirmed OPSCC who were treated between January 2017 and January 2024 at Shanxi Province Cancer Hospital and Shanxi Bethune Hospital.

#### 2.1.2 Inclusion criteria

Eligible patients met the following criteria: A. Pathologically confirmed primary OPSCC with complete medical records; B. Completion of the standard diagnostic and treatment procedures; C. No concomitant malignant tumors during the study period; D. Availability of biopsy or postoperative pathological specimens; E. Comprehensive and complete follow-up data; F. Full understanding by the patients and their families of the multimodal treatment plan and postoperative follow-up requirements for OPSCC, with signed informed consent.

#### 2.1.3 Follow-up

The first follow-up visit was conducted one month after treatment completion. Subsequently, follow-up was scheduled every 3 months within the first year, every 6 months for the next five years, and annually thereafter. The final follow-up date for this study was September 1, 2024.

#### 2.1.4 Study endpoint and grouping

The primary endpoint of this study was overall survival (OS), defined as the time from initial diagnosis to death from any cause or the date of the last follow-up.

Patients were grouped based on p16 expression status. Age was dichotomized according to the median value of 58 years. Primary tumor subsites were classified into two categories based on the degree of lymphoid tissue enrichment. Treatment modality was grouped into surgical and non-surgical categories. The smoking index was calculated using pack-years (PY), defined as the number of cigarettes smoked per day divided by 20 and multiplied by the number of smoking years (PY = [cigarettes/day ÷ 20] × years). Patients were grouped based on the median PY value of 10. Tumor staging was determined according to the AJCC 8th edition TNM classification system.

### 2.2 P16 immunohistochemical staining

Formalin-fixed paraffin-embedded (FFPE) tissue blocks were sectioned into 4 μm-thick slices and baked at 72°C for 45 minutes using a drying oven (Model YD-A, Yidi Medical, Jinhua, Zhejiang, China). Immunohistochemical staining was performed using the Bond-III automated stainer (Leica Biosystems, Germany), which conducted deparaffinization, rehydration, antigen retrieval, and antibody incubation steps in sequence.

The primary antibody was a mouse anti-human p16 monoclonal antibody (clone E6H4, catalog no. 705-4713, Roche/Ventana), and the secondary antibody was EnVision FLEX/HRP (catalog no. K800021-2, Agilent/Dako). DAB chromogen solution (catalog no. DAB-0031, Solarbio) was applied for 3 minutes to visualize the immunoreactivity.

After DAB staining, the slides were rinsed with tap water and counterstained with hematoxylin solution (catalog no. HM-0102, Maixin Biotech) for 1 minute, differentiated for 1–2 seconds, and then blued in running water for 10–15 minutes. The slides were subsequently dehydrated through a graded ethanol series (75%, 95%, and 100%) and cleared in xylene I and II using an automated staining machine (Model HRS-14B, Hangu Medical Technology Co., Ltd., Wuhan, China). Coverslipping was completed using an automated coverslipper (CV5030, Leica Biosystems), and neutral resin (catalog no. NM-0055, Maixin Biotech) was used as the mounting medium.

Microscopic evaluation was performed using an Olympus BX53 optical microscope at 200× and 400× magnifications. Suspected positive regions were first screened at 200×, followed by evaluation at 400× magnification. Five high-power fields (HPFs) were assessed per slide, with at least 200 tumor cells per field and a total of ≥1000 tumor cells evaluated.

Scoring Criteria: Immunohistochemical results were independently evaluated by two experienced pathologists. P16 positivity was defined as strong and diffuse nuclear and cytoplasmic staining in ≥70% of tumor cells (classified as HPV-positive). Focal, weak, or less than 70% staining was considered p16-negative (classified as HPV-negative).

### 2.3 Statistical analysis

All statistical analyses were performed using SPSS version 29.0 (IBM Corp., Armonk, NY, USA). Continuous variables were expressed as mean ± standard deviation (SD) or median with interquartile range (IQR), while categorical variables were presented as counts and percentages [n (%)]. Comparisons between groups were conducted using the Chi-square test or Fisher’s exact test, as appropriate.

Kaplan–Meier survival curves were generated to assess OS, and differences between groups were evaluated using the log-rank test. Cox proportional hazards regression models were applied for univariate and multivariate analyses to identify potential prognostic factors influencing OS. A two-sided P value < 0.05 was considered statistically significant.

## 3 Results

### 3.1 General health characteristics and clinicopathological features of patients with OPSCC

The general health characteristics and clinicopathological features of the 120 patients are summarized in **Table 1**.

**Table 1:** Comparison of general health and clinicopathological characteristics between p16-positive and p16-negative OPSCC patients.

Variable		Total n(%)	P16-	P16+	Chi-Square Value	*P*
		120 (100)	51 (42.5)	69 (57.5)		
general health characteristics						
Gender	Male	104 (86.7)	46	58	0.956	P=0.420
	Female	16 (13.3)	5	11		
Age (years)	≤58	64 (53.3)	27	37	0.005	P=1.000
	>58	56 (46.7)	24	32		
Smoking	No	47 (39.2)	19	28	0.206	P=0.923
	PY<10	10 (8.3)	4	6		
	PY≥10	63 (52.5)	28	35		
Alcohol	No	44 (36.7)	21	23	0.777	P=0.445
	Yes	76 (63.3)	30	46		
Clinicopathological characteristics						
sub-site	Tonsil\Base of tongue	90 (75)	33	57	5.013	P=0.033
	Soft palate\posterior pharyngeal wall	30 (25)	18	12		
Tumor Grade	Highly	12 (10)	5	7	12.167	P=0.002
	Moderately	45 (37.5)	28	17		
	Poorly	63 (52.5)	18	45		
Initial Symptom	Pharyngeal Discomfort	79 (65.8)	40	39	6.258	P=0.019
	Neck Mass	41 (34.2)	11	30		
T stage	T1/T2	94 (78.3)	41	53	0.222	P=0.662
	T3/T4	26 (21.7)	10	16		
N stage	N0/N1	97 (80.8)	35	62	8.529	P=0.005
	N2/N3	23 (19.2)	16	7		
Distant Metastasis	No	109 (90.8)	46	63	0.043	P=1.000^*^
	Yes	11 (9.2)	5	6		
TNM stage	I/II	68 (56.7)	6	62	72.825	P=0.000
	III/IV	52 (43.3)	45	7		
Treatment Modality	Non-Surgical	31 (25.8)	14	17	0.121	P=0.833
	Surgical	89 (74.2)	37	52		
Survival status	Alive	93 (77.5)	32	61	11.074	P=0.001
	Dead	27 (22.5)	19	8		

T, tumor size; N, regional lymph node involvement; M, distant metastasis; TNM stage, tumor–node–metastasis stage according to the AJCC 8th edition.

* Indicates the use of Fisher’s exact test.

The most common anatomical sub-sites of OPSCC were the tonsils and base of the tongue (90 cases, 75%), where the proportion of p16-positive cases was significantly higher compared to other sub-sites (63.3%, P=0.033). Most patients exhibited poorly differentiated pathology (63 cases, 52.5%), with the proportion of p16-positive cases reaching 71.4% in this group (P=0.002). Among OPSCC patients who presented with neck masses as the initial symptom, 73.2% were p16-positive (P=0.019). According to the AJCC 8th edition staging system, 68 patients (56.7%) were categorized as stage I/II, while 52 patients (43.3%) were classified as stage III/IV. The p16 status had no statistically significant impact on the T stage or M stage at diagnosis. However, 62 patients (89.9%, P=0.005) with p16-positive status were classified as N0/N1. In early-stage patients (stages I/II), the p16-positive rate was 91.2% (P=0.000), indicating that p16 positivity was associated with a significant downstaging of the tumor. p16 status was not related to the choice of treatment modality.

### 3.2 Survival analysis: risk factors affecting the prognosis of OPSCC patients

#### 3.2.1 Univariate and multivariate COX regression

The follow-up duration for the 120 patients ranged from 1 to 223 months, with a median follow-up time of 13.5 months. Univariate Cox regression analysis (**Table 2**) showed that alcohol consumption (HR = 2.403, 95% CI: 1.101–5.241, P=0.028), advanced TNM stage (stage III/IV) (HR=3.007, 95% CI: 1.333–6.783, P=0.008), and p16 positivity (HR=0.343, 95% CI: 0.150–0.783, P=0.011) were significantly associated with OS. Variables with P < 0.1 in univariate analysis were included in the multivariate Cox regression model (**Table 2**). In the multivariate analysis, none of the variables reached statistical significance; however, p16 positivity remained marginally associated with improved OS (HR = 0.457, 95% CI: 0.184–1.130, P = 0.090).

**Table 2:** Univariate and Multivariate Cox Regression Analysis of Factors Associated with Overall Survival in Patients with OPSCC.

Variables		Univariate analysis			Multivariate analysis		
		HR	(95% CI)	*P*	HR	(95% CI)	*P*
Gender	Female	0.46	(0.109-1.943)	0.291			
Age (years)	>58	1.04	(0.485-2.229)	0.92			
Smoking	PY=0	1			1		
	PY≥10	2.22	(0.914-5.394)	0.078	1.806	(0.568-5.741)	0.316
	PY<10	2.677	(0.536-13.378)	0.23	2.01	(0.395-10.22)	0.401
Alcohol	Yes	2.403	(1.101-5.241)	0.028	1.755	(0.615-5.007)	0.293
Initial Symptom	Neck Mass	0.748	(0.327-1.71)	0.491			
Tumor Grade	Highly	1			1		
	Poorly	0.401	(0.138-1.169)	0.094	0.409	(0.131-1.276)	0.124
	Moderately	0.609	(0.211-1.764)	0.361	0.616	(0.207-1.827)	0.382
T stage	T3/T4	1.175	(0.439-3.145)	0.748			
N stage	N2/N3	1.451	(0.634-3.322)	0.378			
TNM stage	III/IV	3.007	(1.333-6.783)	0.008	1.961	(0.665-5.779)	0.222
sub-site	Soft palate posterior pharyngeal wall	1.42	(0.648-3.109)	0.381			
Treatment Modality	Surgical treatment	2.15	(0.742-6.232)				
Distant Metastasis	Yes	0.991	(0.339-2.895)	0.987			
p16 Expression	P16+	0.343	(0.15-0.783)	0.011	0.457	(0.184-1.13)	0.09

#### 3.2.2 Kaplan-Meier survival analysis

##### 3.2.2.1 Combined analysis of p16 status and tumor subsite

**Figure 1:**
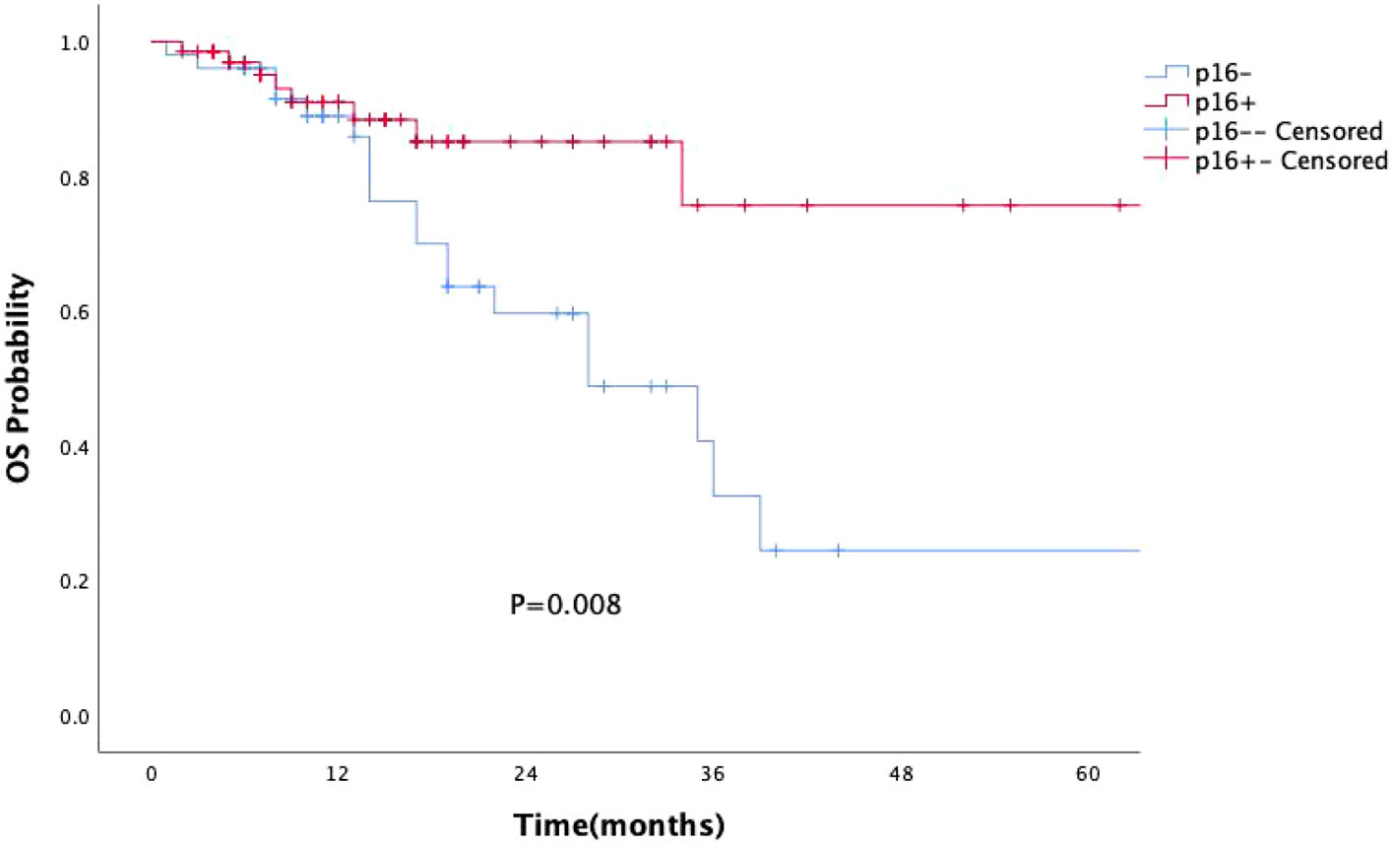
Kaplan-Meier survival curves showing the impact of p16 status on the prognosis of OPSCC patients. p16 positivity is associated with significantly improved prognosis in patients with OPSCC (P=0.008).

**Figure 2:**
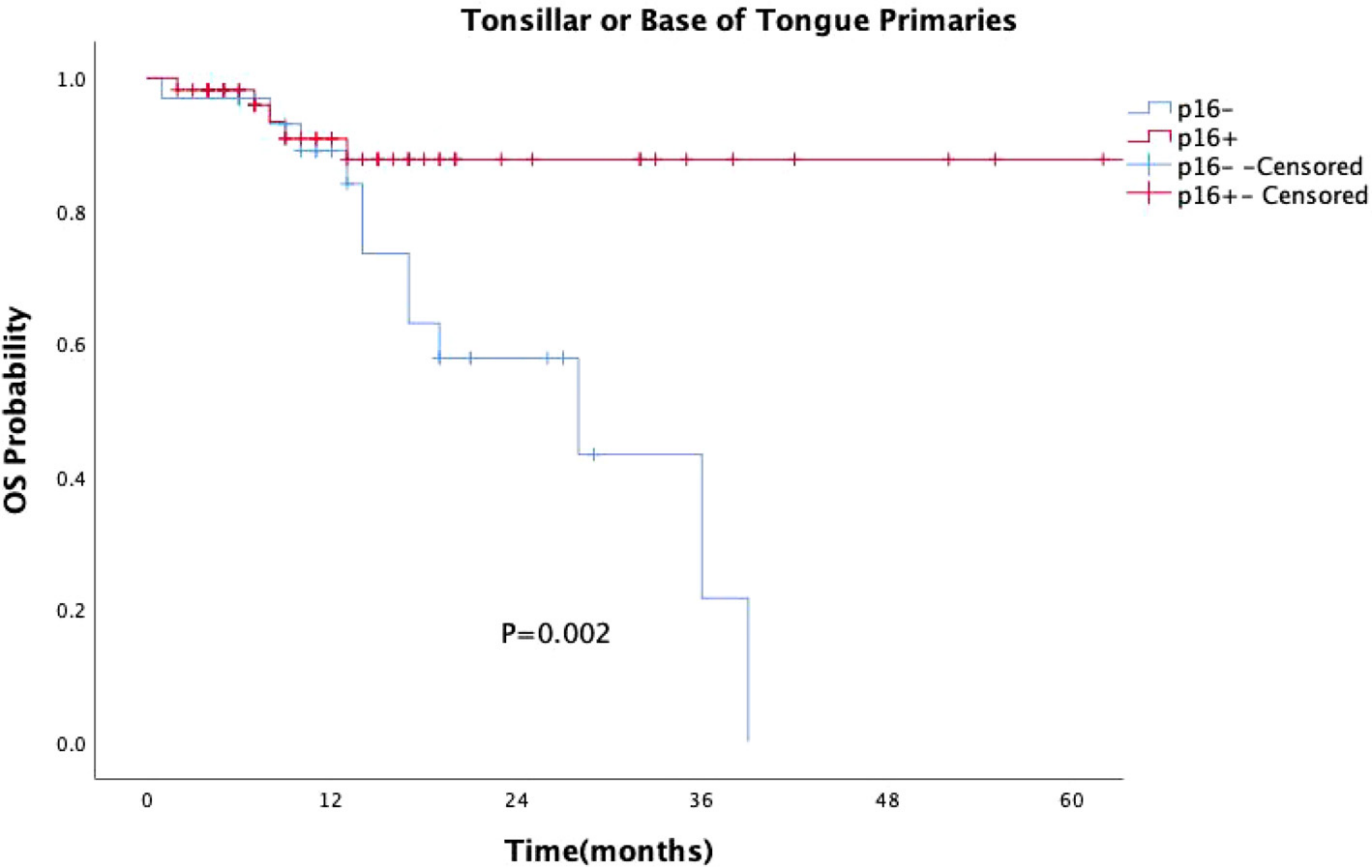
Kaplan-Meier survival curves for OPSCC originating from the tonsil and base of the tongue with different p16 statuses. p16-positive status significantly extends survival in patients with OPSCC originating from the tonsil and base of the tongue (P = 0.002).

**Figure 3:**
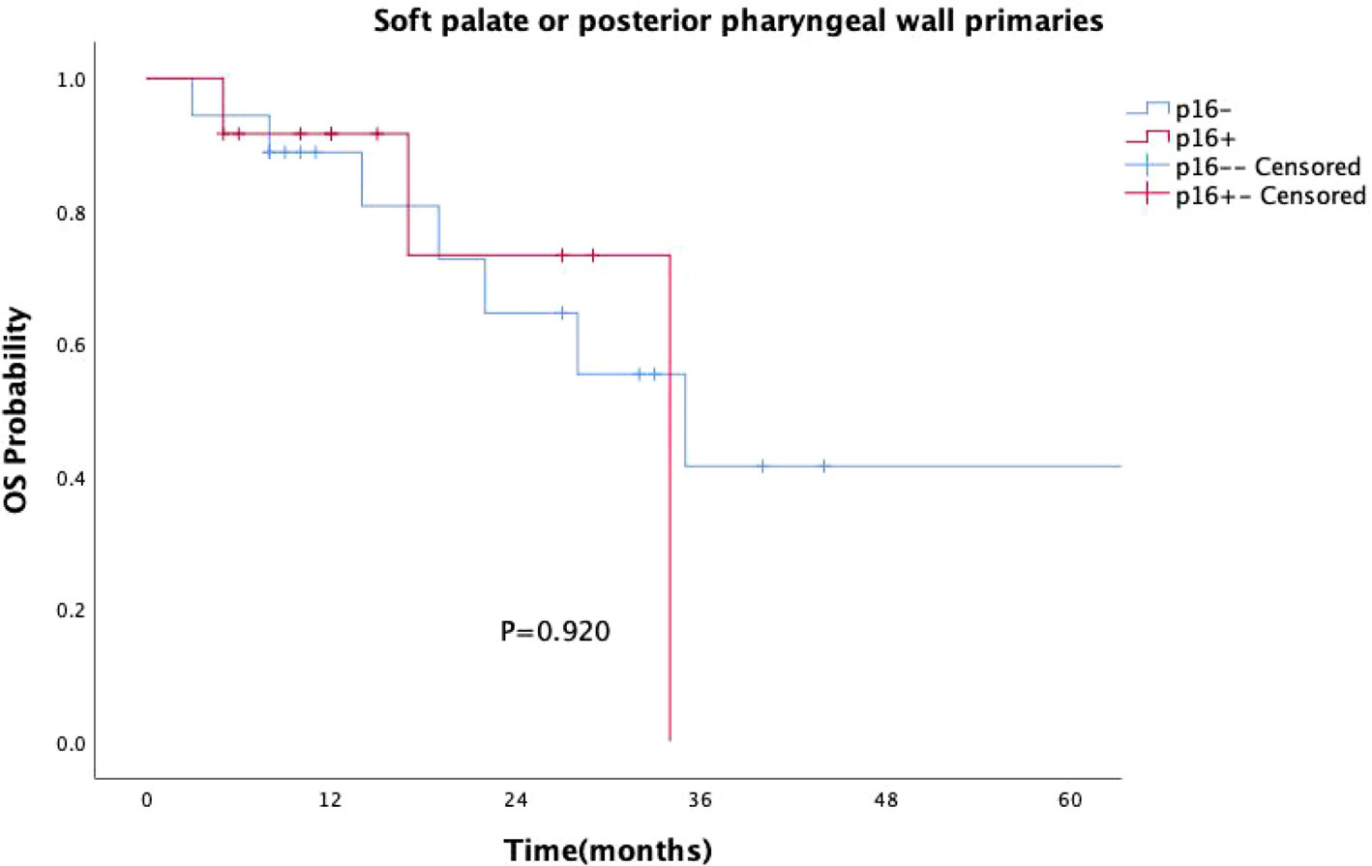
Kaplan–Meier survival curves for OPSCC originating from the soft palate or posterior pharyngeal wall with different p16 statuses. p16-positive status did not significantly improve survival in patients with OPSCC from these subsites (P = 0.920).

p16 status was identified as a significant prognostic factor (P = 0.008, **Figure 1**). Kaplan-Meier survival analysis was conducted with both p16 status and the primary tumor sub-site as prognostic factors. For patients with OPSCC originating from the tonsils and base of the tongue, 57 were p16-positive and 33 were p16-negative. p16 positivity was associated with a significantly reduced mortality rate (P = 0.002, **Figure 2**). However, when the primary tumor was located in the soft palate or posterior pharyngeal wall, there was no significant difference in prognosis between the p16-positive and p16-negative groups (P = 0.920, **Figure 3**).

##### 3.2.2.2 Combined analysis of p16 status and alcohol consumption

**Figure 4:**
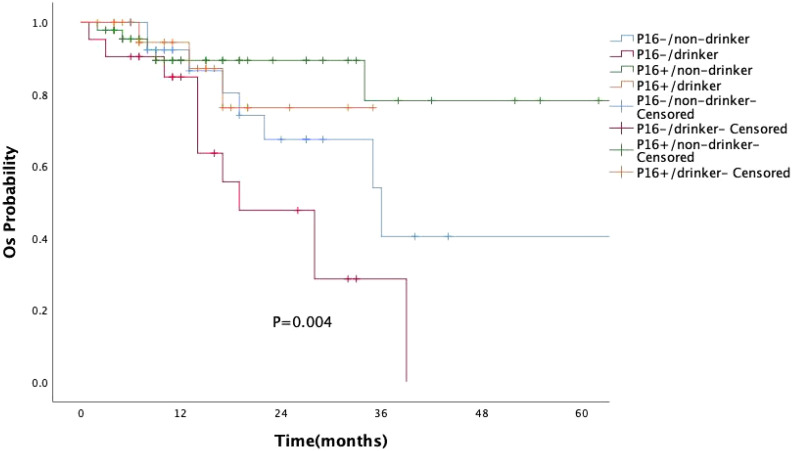
Kaplan–Meier curves of overall survival (OS) based on combined p16 expression and alcohol consumption status. The survival difference among the four groups was statistically significant (P = 0.004, log-rank test). Patients with p16-positive/non-drinker status showed the best prognosis, while those with p16-negative/drinker status had the poorest overall survival.

A subgroup survival analysis was also conducted by combining p16 expression and alcohol consumption (**Figure 4**). Patients were categorized into four groups: p16-positive/non-drinker(n=46), p16-positive/drinker (n=23), p16-negative/non-drinker(n=30), p16-negative/drinker(n=21). The Kaplan–Meier survival curves showed significant differences among the four groups (P =0.004). The best survival outcome was observed in the p16-positive/non-drinker group, whereas the p16-negative/drinker group had the poorest prognosis. Notably, regardless of alcohol consumption status, the survival curves of the two p16-positive groups consistently remained above those of all four groups.

#### 3.2.3 Treatment modalities

The treatment modalities of 120 patients were divided into two groups based on whether they underwent surgery. A total of 89 patients (74.2%) received surgical treatment, including surgery alone (12 patients), postoperative concurrent chemoradiotherapy (18 patients), postoperative radiotherapy alone (47 patients), or postoperative chemotherapy alone (12 patients). A total of 31 patients (25.8%) received non-surgical treatment, including concurrent chemoradiotherapy (17 patients), radiotherapy (4 patients) or chemoradiotherapy (3 patients) alone, and no treatment (7 patients).

**Figure 5:**
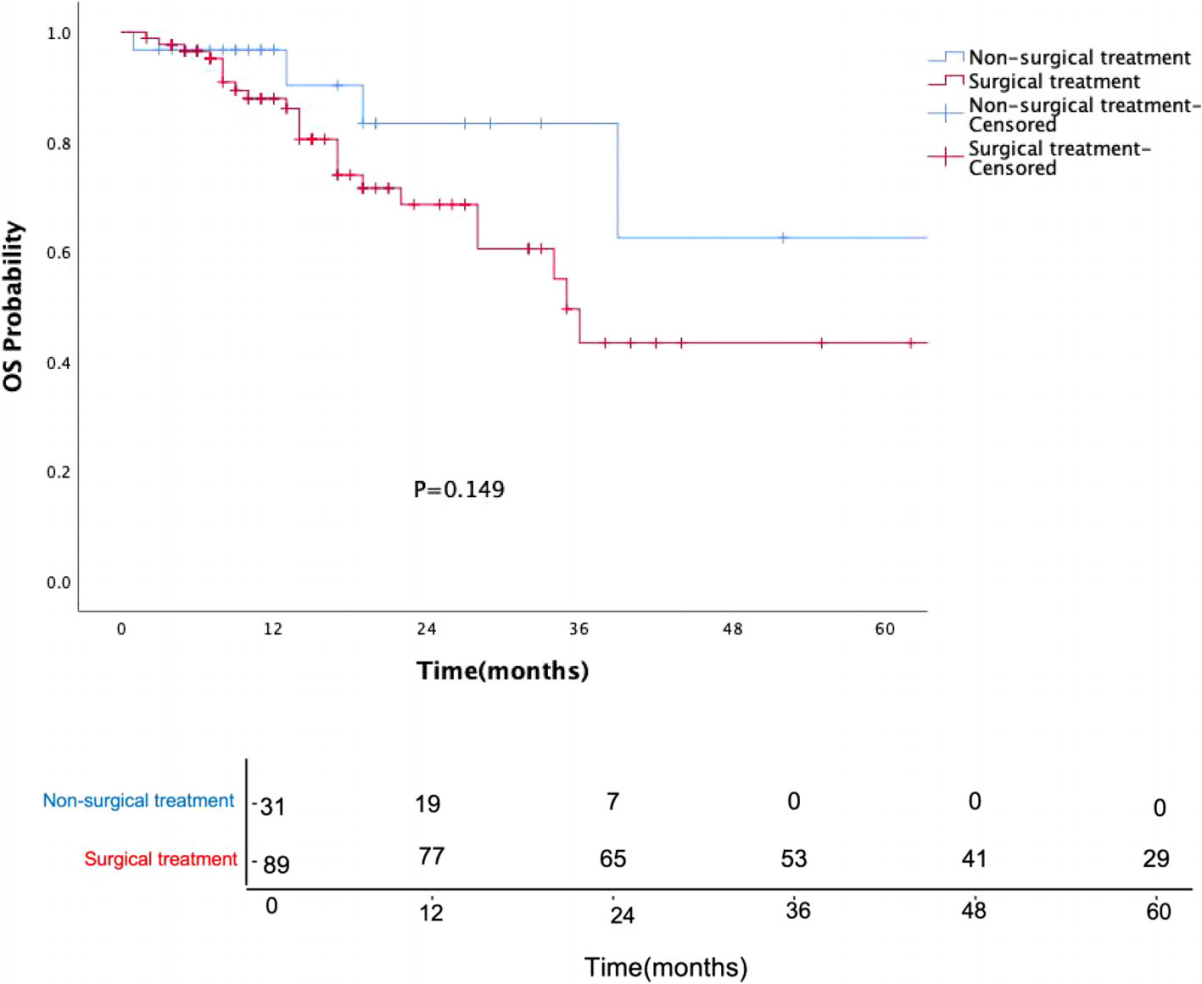
Kaplan-Meier survival curves for OPSCC patients with or without surgery. Numbers indicate the number of surviving patients at each time point. Surgical treatment did not significantly affect prognosis (P = 0.149).

**Figure 6:**
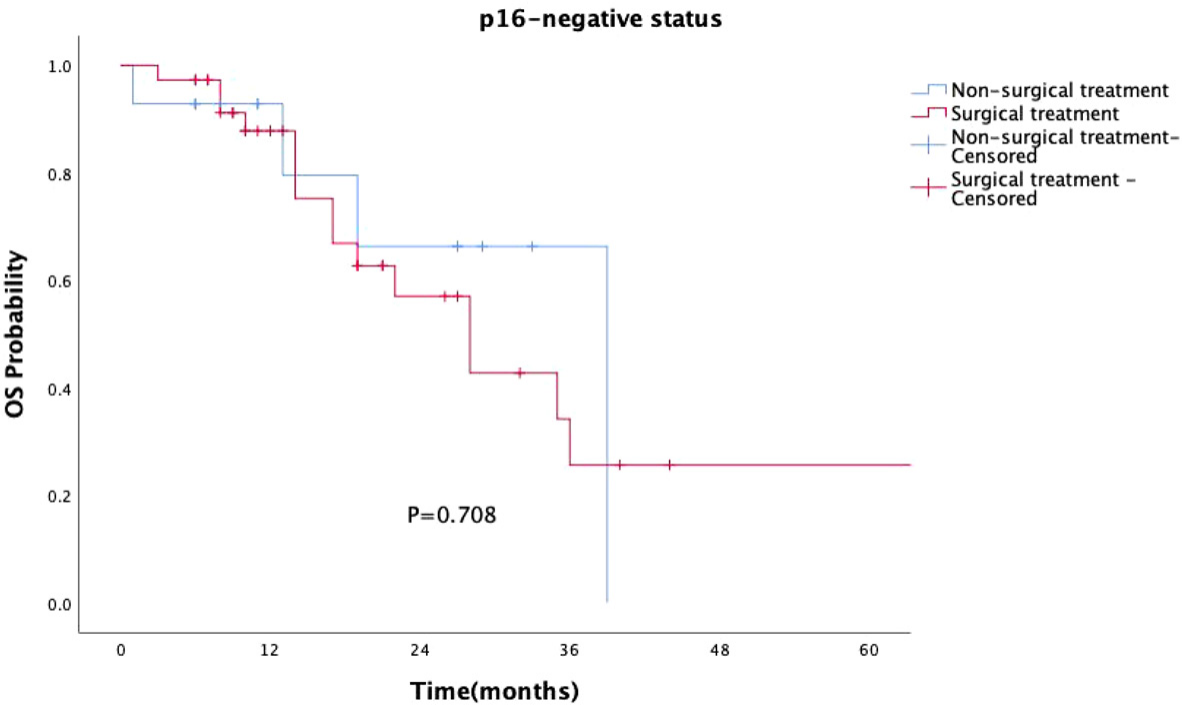
Kaplan-Meier survival curves for p16-negative OPSCC patients with or without surgery. In p16-negative patients, surgical treatment did not significantly impact prognosis (P = 0.708).

**Figure 7:**
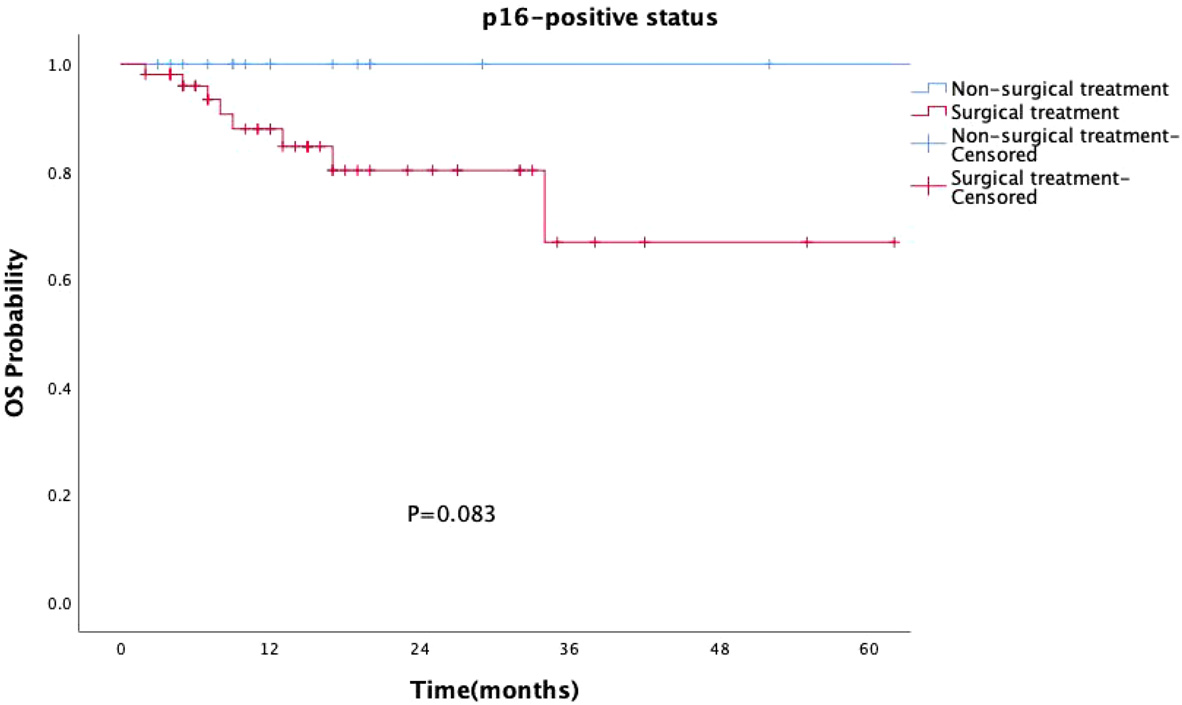
Kaplan-Meier survival curves for p16-positive OPSCC patients with or without surgery. Although the impact of surgery on prognosis in the p16-positive group was not statistically significant (P = 0.083), the survival curves began to show a noticeable divergence approximately one year after treatment, with the surgical group exhibiting a worse prognosis.

Surgical treatment had no impact on prognosis (P = 0.149, **Figure 5**), and this result was validated in the p16-negative group (P = 0.708, **Figure 6**). Although there was no statistically significant impact of surgery on prognosis in the p16-positive group (P = 0.083, **Figure 7**), the survival curves showed a noticeable separation trend after about one year of treatment, with the surgical group exhibiting a worse prognosis.

## 4 Discussion

HPV is a family of sexually transmitted viruses that affect mucosal and skin epithelia (9) and has been identified as a major risk factor for cervical cancer (10). Currently, the incidence of HPV-positive OPSCC in the U.S. and U.K. has surpassed that of cervical cancer (11). The recognition of the association between HPV and oropharyngeal cancer, along with the increasing incidence of HPV-OPSCC, has significantly influenced the screening, diagnosis, and treatment strategies for this disease. Immunohistochemical detection of the p16 protein has been validated as a surrogate marker for HPV-related cancers and is strongly correlated with HPV DNA (12, 13). The 8th edition of the AJCC staging system introduced a distinct staging framework for p16-overexpressing (HPV-associated) OPSCC, acknowledging the favorable prognosis of this subtype (14). Despite the better response to chemoradiotherapy and the improved prognosis associated with HPV positivity, the rising incidence remains a concern. Over the past decade, the incidence of HPV-OPSCC in China has been relatively low (11%-21.28%) (15–17). However, recent data from Fudan University Cancer Center, covering patients from 2019 to 2022, show that the HPV-positive rate has surged to 60% (18). In our study, the number of p16-positive cases among 120 patients reached 69 (57.5%), which is consistent with these findings.

The biological behavior of HPV-OPSCC differs significantly from oropharyngeal cancer driven by traditional risk factors (11, 19). Among the 120 patients in our study, p16 overexpression was not associated with smoking or alcohol consumption, although a history of these habits remained linked to a worse prognosis (20). By combining p16 expression with alcohol consumption status, we found that patients with p16-negative tumors and a history of alcohol use had the poorest prognosis. In contrast, the survival curves of both p16-positive groups consistently remained above those of all four subgroups. These findings suggest that, in oropharyngeal squamous cell carcinoma, p16 status may have a greater impact on prognosis than alcohol consumption. In recent years, the disease burden of OPSCC has increasingly shifted towards older males (21, 22), with a study observing a rapid rise in incidence among white males aged 65 years and older, and nearly 10% of cases occurring in individuals aged 70 and above (11). In our study, although age and gender were not associated with p16 overexpression, approximately 86.7% of the patients were male, with a median diagnosis age of 58 years, and 17.5% were aged 70 years or older, which aligns with the aforementioned research.

OPSCC primarily arises in the tonsils, the base of the tongue, the soft palate, and the posterior pharyngeal wall. Histologically, the mucosal invaginations of the tonsils and base of the tongue form crypts lined with specialized epithelium, known as the “lymphoepithelial region,” which has been shown to provide a favorable environment for HPV-driven tumorigenesis. Conversely, the soft palate and posterior pharyngeal wall are lined with non-keratinized stratified squamous epithelium, referred to as the “non-lymphoepithelial region,” similar to the oral mucosa, which may act as a barrier to HPV infection (23). In our cohort, the most common primary anatomical subsites were the tonsils and the base of the tongue (90 cases, 75%), with a higher proportion of p16 positivity compared to other sites (63.3%, p=0.033). Previous research has indicated that squamous cell carcinomas originating from tonsillar tissue predominantly exhibit non-keratinizing and basaloid histopathology, whereas those arising from non-tonsillar tissues are characterized by keratinizing and non-basaloid appearances (24). Tumors from these distinct anatomical regions may represent different clinical and prognostic entities (25). Additionally, OPSCC originating in the “lymphoepithelial region” tends to metastasize early to lymph nodes, which are often larger (26). In our study, poorly differentiated squamous cell carcinoma was more common in p16-positive patients (65.2%, P=0.002), and a higher proportion of patients with p16 positivity presented with neck masses as the initial symptom (73.2%, P=0.019). Based on these findings, some researchers (27) have proposed that, in cases of cervical metastatic squamous cell carcinoma with an unknown primary site, the tonsils and base of the tongue should be prioritized as potential sites for thorough evaluation.

As research progresses, more studies have suggested that the impact of HPV on survival in OPSCC patients differs between “lymphoepithelial” and “non-lymphoepithelial” subsites (28–30). Our study similarly found that p16-positive patients had significantly better prognoses than p16-negative patients (P=0.008). Specifically, for OPSCC originating from the tonsils and base of the tongue, p16 positivity continued to confer a survival advantage (P=0.002). However, for patients with primary tumors located in the soft palate and posterior pharyngeal wall, there was no statistically significant difference in survival between p16-positive patients and p16-negative patients (P=0.920). The prognostic value of HPV infection for OPSCC patients with primary tumors in the soft palate and posterior pharyngeal wall appears less reliable, raising questions about the applicability of the current AJCC staging system to these sites of squamous cell carcinoma (31). Thus, caution should be exercised when considering de-escalation treatment in such cases.

Currently, clinical guidelines have not differentiated treatment strategies for oropharyngeal cancer based on HPV status, and de-escalation treatment remains in the clinical research phase. In our study, the choice of treatment was independent of HPV status and tumor stage, with post-operative radiotherapy being the primary treatment option (I/II stage: 23 cases, 33.8%; III/IV stage: 24 cases, 46.2%). Surgical procedures included primary tumor resection (with or without neck dissection) or isolated neck dissection, all performed as open surgeries without using transoral robotic surgery. Surgery did not statistically improve the prognosis of oropharyngeal cancer patients (P=0.149), particularly in the p16-positive group, where the prognosis of surgically treated patients was slightly worse than that of the non-surgical group, especially after one year of treatment. Traditional open surgery is not conducive to preserving oropharyngeal function and improving the quality of life, especially for HPV-OPSCC patients, who should consider this treatment option with caution.

## 5 Conclusions

In conclusion, our study demonstrates that HPV-OPSCC has unique biological and clinical characteristics. With economic development, the incidence of HPV-OPSCC in China has rapidly increased, and in the less-developed Shanxi region, the proportion of HPV-positive patients among OPSCC cases has surpassed that of oropharyngeal cancer caused by smoking and alcohol consumption. De-escalation treatment for HPV-OPSCC has not yet been implemented, but aggressive treatment without considering HPV status does not benefit patients. Not all HPV-positive OPSCC cases have a favorable prognosis, and the anatomical subsite of the primary tumor should be carefully considered when planning de-escalation treatment in the future. Identifying potential biomarkers for poor prognosis in HPV-positive OPSCC and combining specific biomarkers to achieve precise risk stratification for OPSCC patients will be crucial in selecting individualized treatment strategies.

This study is a retrospective analysis with a relatively small sample size, particularly for patients with primary tumors located in the soft palate or posterior pharyngeal wall, who were significantly fewer than those with tonsillar or base of tongue cancers, which may have introduced selection bias. In addition, the follow-up period was relatively short, and post-treatment quality of life was not assessed, representing certain limitations. In the future, prospective multi-center studies with larger sample sizes, balanced inclusion of patients across different primary tumor sites, extended follow-up durations, and incorporation of multidimensional outcomes such as quality of life assessments are warranted.

## Data Availability

The raw data supporting the conclusions of this article will be made available by the authors, without undue reservation.

## References

[1] Sung H Ferlay J Siegel RL Laversanne M Soerjomataram I Jemal A. Global cancer statistics 2020: GLOBOCAN estimates of incidence and mortality worldwide for 36 cancers in 185 countries. CA Cancer J Clin. (2021) 71:209–49. doi: 10.3322/caac.21660 33538338

[2] Carlander AF Jakobsen KK Bendtsen SK Garset-Zamani M Lynggaard CD Jensen JS. A contemporary systematic review on repartition of HPV-positivity in oropharyngeal cancer worldwide. Viruses. (2021) 13:1326. doi: 10.3390/v13071326 34372532 PMC8310083

[3] Nguyen AT Luu M Mallen-St Clair J Mita AC Scher KS Lu DJ. Comparison of survival after transoral robotic surgery vs nonrobotic surgery in patients with early-stage oropharyngeal squamous cell carcinoma. JAMA Oncol. (2020) 6:1555–62. doi: 10.1001/jamaoncol.2020.3172 PMC7441465 32816023

[4] Nichols AC Theurer J Prisman E Read N Berthelet E Tran E. Radiotherapyversus transoral robotic surgery and neck dissectionfor oropharyngeal squamous cell carcinoma (ORA-TOR): an open-label, phase 2, randomised tria. Lancet Oncol. (2019) 20:1349–59. doi: 10.1016/S1470-2045(19)30410-3 31416685

[5] Palma DA Prisman E Berthelet E Tran E Hamilton S Wu J. Assessmentof toxic effects and survival in treatment deescalation with radiotherapy vs transoral surgery for HPV-associated oropharyngeal squamous cell carcinoma: the ORATOR2 phase 2 randomized clinical trial. JAMA Oncol. (2022) 8:1–7. doi: 10.1001/jamaoncol.2022.0615 PMC9052108 35482348

[6] Yom SS Torres-Saavedra P Caudell JJ Waldron JN Gillison ML Xia P. Reduced-dose radiation therapy for HPV-associated oropharyngeal carcinoma(NRG oncology HN002). J Clin Oncol. (2021) 39:956–65. doi: 10.1200/JCO.20.03128 PMC8078254 33507809

[7] Guo T Kang SY Cohen EEW. Current perspectives on recurrent HPV-mediated oropharyngeal cancer. Front Oncol. (2022) 12:966899. doi: 10.3389/fonc.2022.966899 36059671 PMC9433540

[8] Guo T Zamuner F Ting S Chen L Rooper L Tamayo P. Clinical and genomic characterization of chemoradiation-resistant HPV-positive oropharyngeal squamous cell carcinoma. Front Oncol. (2024) 14:1336577. doi: 10.3389/fonc.2024.1336577 38505587 PMC10949886

[9] Pytynia KB Dahlstrom KR Sturgis EM. Epidemiology of HPV-associated oropharyngeal cancer. Oncol. (2014) 50:380–6. doi: 10.1016/j.oraloncology.2013.12.019 PMC4444216 24461628

[10] Williamson A Jashek-Ahmed F Hardman J Paleri V. Functional and quality-of-life outcomes following salvage surgery for recurrent squamous cell carcinoma of the head and neck: A systematic review and meta-analysis. Eur Arch Oto-Rhino-Laryngol. (2023) 280:4597–618. doi: 10.1007/s00405-023-08056-z 37329358

[11] Lechner M Liu J Masterson L Fenton TR. HPV-associated oropharyngeal cancer: epidemiology, molecular biology and clinical management. Nat Rev Clin Oncol. (2022) 19:306–27. doi: 10.1038/s41571-022-00603-7 PMC8805140 35105976

[12] Salazar CR Anayannis N Smith RV Wang Y Haigentz M Jr Garg M. Combined p16 and human papillomavirus testing predicts head and neck cancer survival. Int J Cancer. (2014) 135:2404–12. doi: 10.1002/ijc.v135.10 PMC4159440 24706381

[13] Augustin JG Lepine C Morini A Brunet A Veyer D Brochard C. HPV detection in head and neck squamous cell carcinomas: what is the issue? Front Oncol. (2020) 10:1751. doi: 10.3389/fonc.2020.01751 33042820 PMC7523032

[14] Lydiatt WM Patel SG O’Sullivan B Brandwein MS Ridge JA Migliacci JC. Head and Neck cancers-major changes in the American Joint Committee on cancer eighth edition cancer staging manual. CA Cancer J Clin. (2017) 67:122–37. doi: 10.3322/caac.21389 28128848

[15] Wang Z Xia R-H Ye D-X Li J. Human papillomavirus 16 Infection and TP53 mutation: two distinct pathogeneses for oropharyngeal squamous cell carcinoma in an Eastern Chinese population. PloS One. (2016) 11:1:e016449. doi: 10.1371/journal.pone.0164491 PMC5066983 27749915

[16] Meng HX Miao SS Chen K Li HN Yao G Geng J. Association of p16 as Prognostic Factors for Oropharyngeal Cancer: Evaluation of p16 in 1470 Patients for a 16 Year Study in Northeast China. BioMed Res Int. (2018) 2018:1–8. doi: 10.1155/2018/9594568 PMC6166388 30310820

[17] Xu S Sun B Zhou R Shi C Han Y Li J. Evaluation of p16 as a surrogate marker for transcriptionally active human papillomavirus status of oropharyngeal squamous cell carcinoma in an eastern Chinese population. Surg Med Pathol Radiol. (2020) 129:236–45. doi: 10.1016/j.oooo.2019.11.008 31987673

[18] Wei Y Xu T Li C. CD161 characterizes an inflamed subset of cytotoxic T lymphocytes associated with prolonged survival in human papillomavirus driven oropharyngeal cancer. Cancer Immunol Res. (2023) 11:306–19. doi: 10.1158/2326-6066.CIR-22-0454 PMC9975669 36633583

[19] Chaturvedi AK Freedman ND Abnet CC. The evolving epidemiology of oral cavity and oropharyngeal cancers. Cancer Res. (2022) 82:2821–3. doi: 10.1158/0008-5472.CAN-22-2124 35971675

[20] Chen SY Massa S Mazul AL Kallogjeri D Yaeger L Jackson RS. The association of smoking and outcomes in HPV-positive oropharyngeal cancer: A systematic review. Am J Otolaryngol. (2020) 5):41. doi: 10.1016/j.amjoto.2020.102592 32521295

[21] Kreimer AR Chaturvedi AK Alemany L Anantharaman D Bray F Carrington M. Summary from an international cancer seminar focused on human papillomavirus (HPV)-positive oropharynx cancer, convened by scientists at IARC and NCI. Oncol. (2020) 108:104736. doi: 10.1016/j.oraloncology.2020.104736 PMC7909748 32502860

[22] Tota JE Best AF Zumsteg ZS Gillison ML Rosenberg PS Chaturvedi AK. Evolution of the oropharynx cancer epidemic in the United States: moderation of increasing incidence in younger individuals and shift in the burden to older individuals. J Clin Oncol. (2019) 37:1538–46. doi: 10.1200/JCO.19.00370 PMC6599405 31026209

[23] Haeggblom L Attoff T Hammarstedt-Nordenvall L Näsman A. Human papilloma virus and survival of patients per histological subsite of tonsillar squamous cell carcinoma. Cancer Med. (2018) 7:1717–22. doi: 10.1002/cam4.2018.7.issue-5 PMC5943436 29573210

[24] Gelwan E Malm I-J Khararjian A Fakhry C Bishop JA Westra WH. Nonuniform distribution of high-risk human papillomavirus in squamous cell carcinomas of the oropharynx. Am J Surg Pathol. (2017) 41:1722–8. doi: 10.1097/PAS.0000000000000929 28877058

[25] Fossum CC Chintakuntlawar AV Price DL Garcia JJ. Characterization of the or opharynx: anatomy, histology, immunology, squamous cell carcinoma and surgical resection. Histopathology. (2017) 70:1021–9. doi: 10.1111/his.2017.70.issue-7 27926789

[26] Stjernstrøm KD Jensen JS Jakobsen KK Grønhøj C von Buchwald C. Current status of human papillomavirus positivity in oropharyngeal squamous cell carcinoma in Europe: a systematic review. Acta Oto Laryngologica. (2019) 139:1112–6. doi: 10.1080/00016489.2019.1669820 31560260

[27] Wen SX Wen KX Zhang YH Zhang L Wang R Wang C. Considerations and clinical recommendations on the progression and management of cervical lymph node metastasis from unknown primary sites. Chin J Otorhinolaryngol Head Neck Surgery. (2024) 59:1389–92. doi: 10.3760/cma.j.cn115330-20240721-00434 39734297

[28] Marklund L Holzhauser S de Flon C Zupancic M Landin D Kolev A. Survival of patients with oropharyngeal squamous cell carcinomas (OPSCC) in relation to TNM 8—Risk of incorrect downstaging of HPV-mediated non-tonsillar, non-base of tongue carcinomas. Eur J Cancer. (2020) 139:192–200. doi: 10.1016/j.ejca.2020.08.003 32951963

[29] Hammarstedt L Holzhauser S Zupancic M Kapoulitsa F Ursu RG Ramqvist T. The value of p16 and HPV DNA in non-tonsillar, non-base of tongue oropharyngeal cancer. Acta Otolaryngol. (2021) 141:89–94. doi: 10.1080/00016489.2020.1813906 32940116

[30] Tham T Wotman M Roche A Kraus D Costantino P. The prognostic effect of anatomic subsite in HPV-positive oropharyngeal squamous cell carcinoma. Am J Otolaryngol. (2019) 40:567–72. doi: 10.1016/j.amjoto.2019.05.006 31113681

[31] Wendt M Hammarstedt-Nordenvall L Zupancic M Friesland S Landin D Munck-Wikland E. Long-term survival and recurrence in oropharyngeal squamous cell carcinoma in relation to subsites, HPV, and p16-status. Cancers (Basel). (2021) 13:2553. doi: 10.3390/cancers13112553 34070952 PMC8196945

